# Juvenile localized scleroderma: review of 44 patients

**DOI:** 10.1186/1546-0096-9-S1-P75

**Published:** 2011-09-14

**Authors:** E Iglesias, J Antón, S Ricart, J Ros, V Torrente, R Bou, MA González, A Vicente

**Affiliations:** 1 Department of Pediatric Infectious Diseases and Immunodeficiencies, Hospital Infantil Virgen del Rocío, Seville, Spain; 2 Pediatric Rheumatology Unit, Hospital Sant Joan de Deu, Esplugues de Llobregat, Barcelona, Spain; 3 Department of Dermatology, Hospital Sant Joan de Deu, Esplugues de Llobregat, Barcelona, Spain

## Introduction

Predominant form of childhood sclerosis is localized scleroderma (LSc) or morphoea. Preliminary classification includes circumscribed morphoea (CM), linear scleroderma (LS), generalized (GM), panclerotic (PM) and mixed morphoea (MM). The major problem in untreated patients is morbidity, so the aim of the therapy is to prevent this.

## Methods

We report data on clinical, laboratory and treatment features in a descriptive observational study of 44 children with LSc assisted at our hospital from January 2000 to November 2010.

## Results

**Figure 1:**
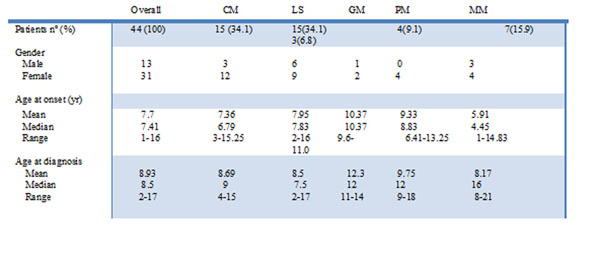
Main demographic features. Abbreviations: circumscribed morphoea (CM), linear scleroderma (LS), generalized (GM), panclerotic (PM) and mixed morphoea (MM).

70.5% of patients were females. CM (34.1%) and LM (34.1%) were the most frequently subtypes. Mean age at first symptoms was 7.7 years (Figure 1). Medium time between first signs/symptoms and diagnosis was 15.42 months. One patient with CM head-neck lesions had Parry-Romberg disease with neurological involvement. 50% of patients with head-neck LM had “coup de sabre” subtype, 25% of that with neurological involvement. One patient had joint movement limitation and another limb dysmetria. 38.6% patients tested positive for ANA, none developed SLE. Scl-70 and anticentromere were negative in all. Drugs most frequently used were topical corticosteroids (61.3%), methotrexate and systemic corticosteroids (38.6%) and topical tacrolimus (36.4%). Time to remission was 6.95 months. 34.09% of patients had clinical relapse, 26.6% of these being on treatment.

## Conclusions

1.- Long time to diagnosis shows the poor recognition of clinical features of LSc and makes difficult an early treatment to prevent disabilities.

2.- Neurological involvement in patients with lesions on head and neck suggests the importance of an exhaustive neurological examination in these patients.

